# Supplementary material for: Wild raccoons (Procyon lotor) as a potential reservoir of cytolethal distending toxin-producing Providencia strains in Japan
Source: Microbiol Spectr. 2025 Feb 25;13(4):e02616-24. doi: 10.1128/spectrum.02616-24 (PMC11960107; doi:10.1128/spectrum.02616-24)
Supplement: Supplemental figures — Figures S1 to S3. [file spectrum.02616-24-s0001.pdf]

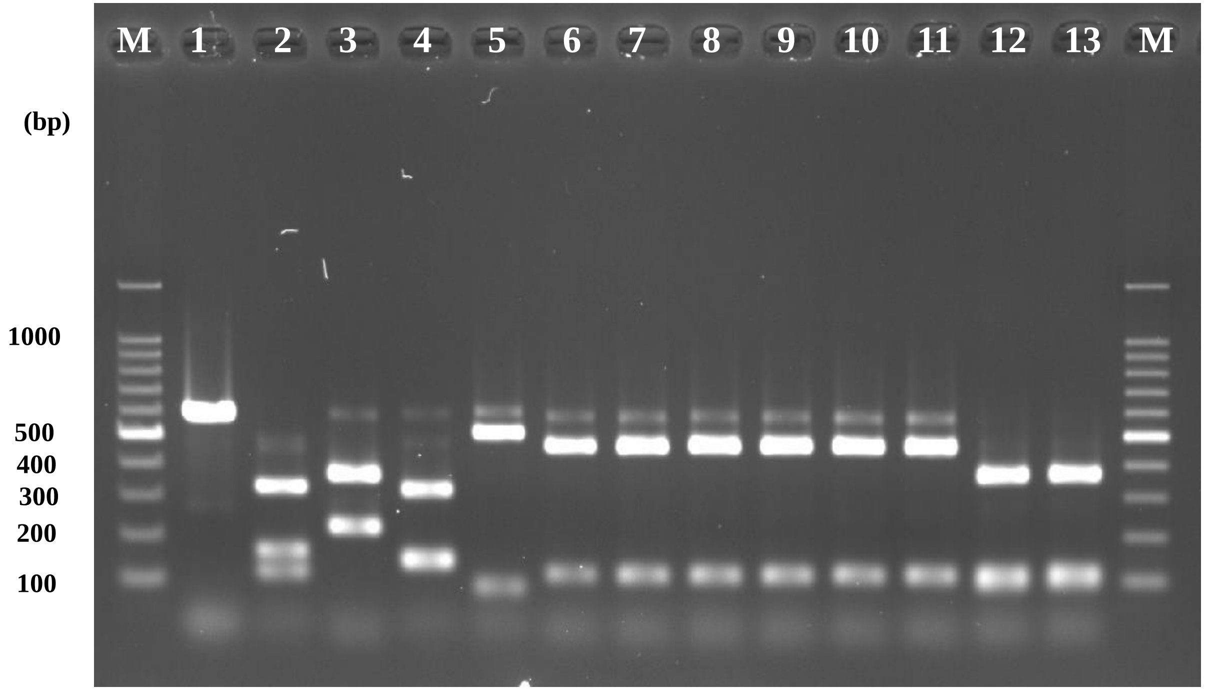

FIG S1 Detection and subtyping of *cdt* genes by PCR-RFLP assay.

Escherichia coli cdtB gene subtypes primer pairs from Hinenoya et al (22) were used to amplify *cdtB* sequences in the strains and PCR products were digested by *Msp*I and resolved in 3% agarose gel (representative image).

M:, 100 bp DNA ladder (Takara); 1, Undigested *cdtB* amplicon; 2, *Eccdt-I* (*E. coli* strain GB1371); 3, *Eacdt* (*E. albertii* strain P2543; 4, *Eccdt-III/V* (*E. coli* strain AH-16); 5, *Eccdt-IV* (*E. coli* strain AH-12); 6, *cdt P. alcalifaciens* AH-31 (clinical strain); 7, *cdt P. rustigianii* JH-1 (clinical strain); 8, *cdt P. alcalifaciens* RAC2295A (raccoon strain); 9, *cdt P. alcalifaciens* RAC2372A (raccoon strain); 10, *cdt P. rustigianii* RAC2084A (raccoon strain); 11, *cdt P. rustigianii* RAC2100A (raccoon strain); 12, Untypable (novel) *cdt P. rettgeri* RAC2130A (raccoon strain); 13, Untypable (novel) *cdt P. rettgeri* RAC2242A (raccoon strain).

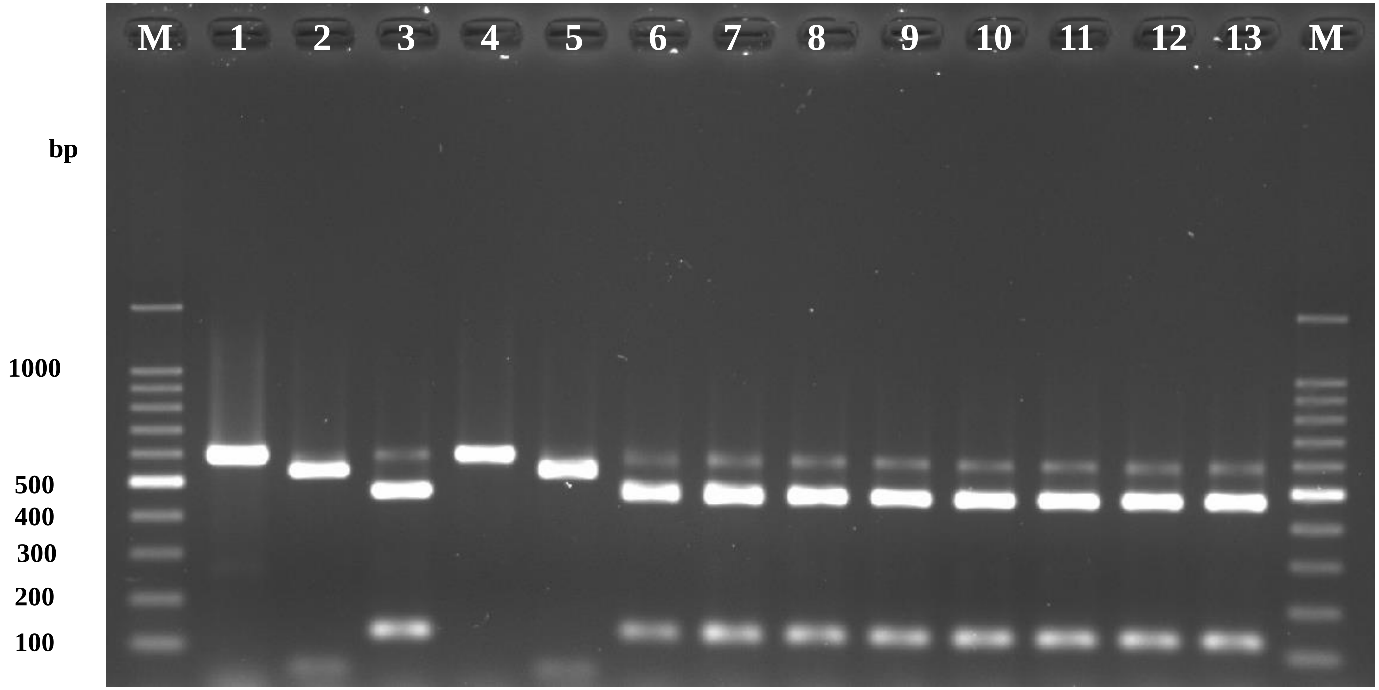

FIG S2 Detection and subtyping of *cdt* genes by PCR-RFLP assay.
Escherichia coli cdtB gene subtypes primer pairs from Hinenoya et al (22) were used to amplify *cdtB* sequences in the strains and PCR products were digested by *Eco*RI/*Eco*RV and resolved in 3% agarose gel (representative image).
M:, 100 bp DNA ladder (Takara); 1, Undigested *cdtB* amplicon; 2, *Eccdt-I* (*E. coli* strain GB1371); 3, *Eacdt* (*E. albertii* strain P2543; 4, *Eccdt-III/V* (*E. coli* strain AH-16); 5, *Eccdt-IV* (*E. coli* strain AH-12); 6, *cdt P. alcalifaciens* AH-31 (clinical strain); 7, *cdt P. rustigianii* JH-1 (clinical strain); 8, *cdt P. alcalifaciens* RAC2295A (raccoon strain); 9, *cdt P. alcalifaciens* RAC2372A (raccoon strain); 10, *cdt P. rustigianii* RAC2084A (raccoon strain); 11, *cdt P. rustigianii* RAC2100A (raccoon strain); 12, (novel) *cdt P. rettgeri* RAC2130A (raccoon strain); 13, (novel) *cdt P. rettgeri* RAC2242A (raccoon strain).

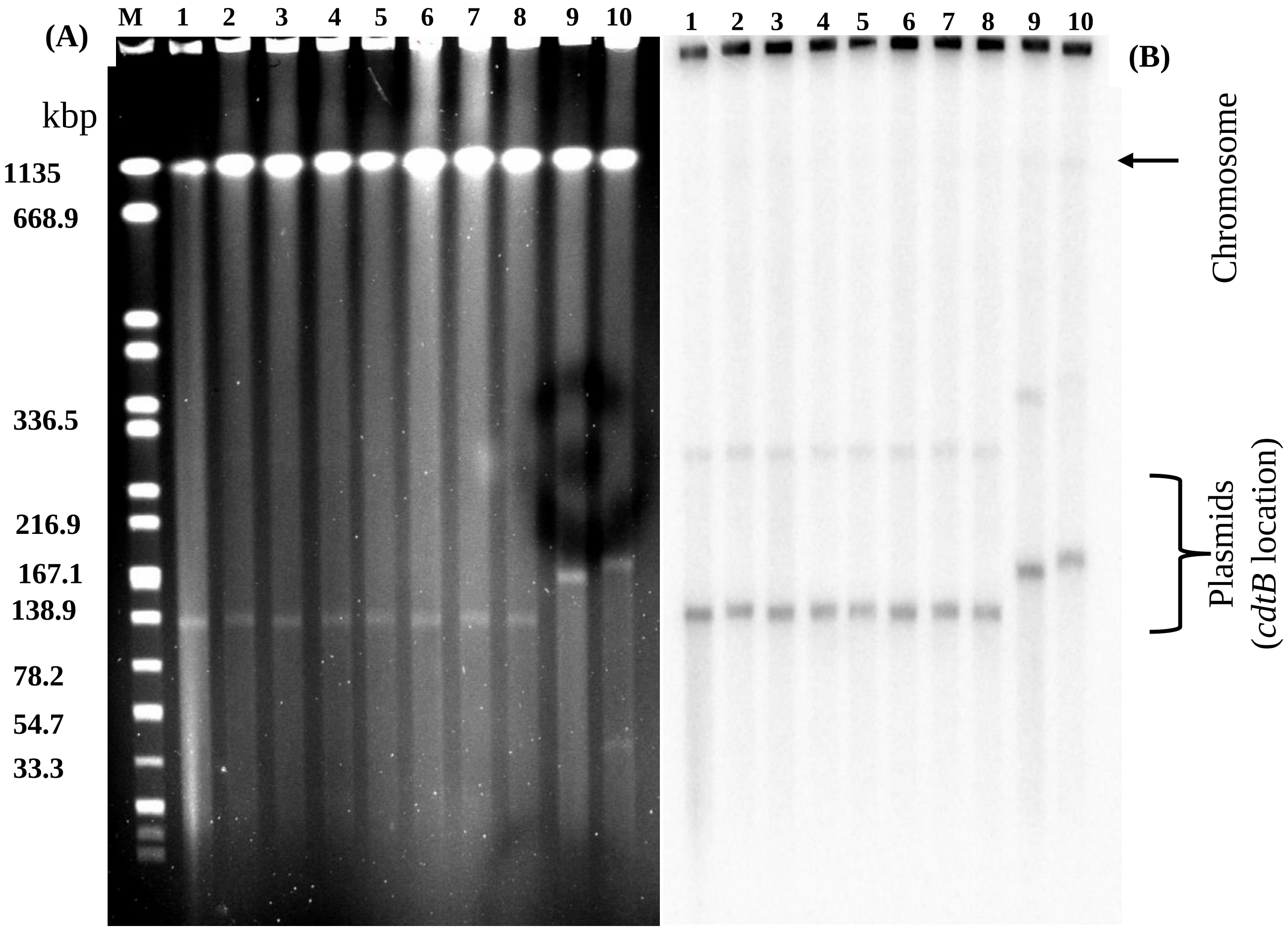

FIG S3 Location of *cdt* genes in the *Providencia* strains by S1-nuclease PFGE and Southern hybridization (representative image). Genomic DNA of the *Providencia* strains was analyzed by S1 nuclease-PFGE (A) and Southern hybridization assays using ^{32}P-labeled *cdtB* gene probe (B). Lanes1-8, *P. rettgeri* strains; lane 9, *P. rustigianii* JH-1 (control 1); lane 10, *P. alcalifaciens* AH-31 (control 2); M, molecular size marker (*Salmonella* Braenderup strain H9812 *Xba*I-digested genomic DNA).
