# Supplementary material for: Wild raccoons (Procyon lotor) as a potential reservoir of cytolethal distending toxin-producing Providencia strains in Japan
Source: Microbiol Spectr. 2025 Feb 25;13(4):e02616-24. doi: 10.1128/spectrum.02616-24 (PMC11960107; doi:10.1128/spectrum.02616-24)
Supplement: Supplemental tables — Tables S1 to S3. [file spectrum.02616-24-s0002.docx]

Table S1. Prototype biochemical patterns of the *Providencia* species.

| S/N | Specie | Ure | Ara | Ino | Rha | Sor | Tre | Raf | Ado | Cit |
| --- | --- | --- | --- | --- | --- | --- | --- | --- | --- | --- |
| 1 | *P. alcalifaciens* | - | - | - | - | - | - | - | + | V |
| 2 | *P. rustigianii* | - | - | - | - | - | - | - | - | V |
| 3 | *P. rettgeri* | + | - | + | + | V | - | V | + | V |
| 4 | *P. stuartii* | V | - | + | - | - | + | - | V | V |
| 5 | *P. vermicola* | + | V | V | - | - | - | V | + | - |
| 6 | *P. heimbachae* | - | - | + | V | - | V | - | - | + |
| 7 | *P. huaxiensis* | + | - | + | - | - | - | - | + | + |

V, variable; Ure, urease; Ara, L-arabinose; Ino, myo-inositol; Rha, L-rhamnose; Sor, D-sorbitol; Tre, trehalose; Raf, raffinose; Ado, adonitol; Cit, citrate

Table S2. Genomic characterization of the representative *Providencia* isolates.

| Number of isolates  (*cdt* genes) | Target genes | | Biochemically identified species |
| --- | --- | --- | --- |
|  | 16S rRNA | *rpoB* |  |
| 24 (+) | *P. alcalifaciens* | *P. alcalifaciens* | *P. alcalifaciens* |
| 10 (-) | “ “ | “ “ | “ “ |
| 6 (+) | *P. rustigianii* | *P. rustigianii* | *P. rustigianii* |
| 2 (-) | “ “ | “ “ | “ “ |
| 8 (+) | *P. rettgeri* | *P. rettgeri* | *P. rettgeri* |
| 4 (-) | “ “ | “ “ | “ “ |
| 5 (-) | *P. stuartii* | *P. stuartii* | *P. stuartii* |
| 2 (-) | *P. heimbachae* | *P. heimbachae* | *P. heimbachae* |
| 2 (-) | *P. vermicola* | *P. vermicola* | *P. vermicola* |
| 2 (-) | *P. huaxiensis* | *P. huaxiensis* | *P. huaxiensis* |

Table S3. List of primers used in this study.

| Primer | Sequence (5’ – 3’) | Target | Reference |
| --- | --- | --- | --- |
| Psp 16S-F3 | CTC TTA GGA GCA AAG CAG | 16S rRNA | present study |
| Psp 16S-R3 | ACC AGT CTT AGA TGC CAT |  |  |
| Pcdt(28-46)-F | ATA GTT GGA GCA TTG ATA C | *cdt* | present study |
| Pcdt(752-733)-R | TGC TAA TCT AGT TCT ATT AG |  |  |
| Cdt-Bcomu1 | TAA ATG GAA TAT ACA TGT CCG | *E. coli* *cdt-IB*, -*IIIB*, -*IVB*, -*VB*  *E. albertii cdtB*, *Providencia cdtB* | 22 |
| Cdt-Bcomu2 | TAA ATG GAA TAT ACA TGT TCG |  |  |
| Cdt-Bcomd | TTT CCA GCT ACT GCA TAA TC |  |  |
| InvF-F | ATG CTG AAT CCT GTT GAA | *invF* | 19 |
| InvF-R | AAT CAC GAG GAG ATA CTC |  |  |
| cJH-1-ATPsyn-F | ACA TCC AGT GCG CAT TCA | *cspaL* | 19 |
| cJH-1-ATPsyn-R | ATG TTC TGG TGT AGT CAC |  |  |
